# Belotecan/cisplatin versus etoposide/cisplatin in previously untreated patients with extensive-stage small cell lung carcinoma: a multi-center randomized phase III trial

**DOI:** 10.1186/s12885-016-2741-z

**Published:** 2016-08-26

**Authors:** In-Jae Oh, Kyu-Sik Kim, Cheol-Kyu Park, Young-Chul Kim, Kwan-Ho Lee, Jin-Hong Jeong, Sun-Young Kim, Jeong-Eun Lee, Kye-Chul Shin, Tae-Won Jang, Hyun-Kyung Lee, Kye-Young Lee, Sung-Yong Lee

**Affiliations:** 1 Department of Internal Medicine, Lung and Esophageal Cancer Clinic, Chonnam National University Hwasun Hospital, 322 Seoyang-ro, Hwasun-eup, Jeonnam 58128 South Korea; 2 Department of Internal Medicine, Yeungnam University Medical Center, Yeungnam University College of Medicine, Daegu, Republic of Korea; 3 Department of Internal Medicine, Chungnam National University Hospital, Chungnam National University School of Medicine, Daejeon, Republic of Korea; 4 Department of Internal Medicine, Yonsei University Wonju Severance Christian Hospital, Yonsei University College of Medicine, Wonju, Republic of Korea; 5 Department of Internal Medicine, Kosin University Gospel Hospital, Kosin University College of Medicine, Busan, Republic of Korea; 6 Department of Internal Medicine, Inje University Busan Paik Hospital, Busan, South Korea; 7 Department of Internal Medicine, Konkuk University Medical Center, Konkuk University School of Medicine, Seoul, Republic of Korea; 8 Department of Internal Medicine, Guro Hospital, Korea University, Seoul, South Korea

**Keywords:** Small cell lung carcinoma, Extensive stage disease, Phase III study, Chemotherapy, First-line, Belotecan

## Abstract

**Background:**

No novel chemotherapeutic combinations have demonstrated superior efficacy to etoposide/cisplatin (EP), a standard treatment regimen for extensive-stage small cell lung carcinoma (ES-SCLC) over the past decade. We aimed to compare the efficacy and safety of belotecan/cisplatin (BP) and EP regimens in chemotherapy- and radiotherapy-naïve patients with previously untreated ES-SCLC.

**Methods:**

We conducted a multi-center, randomized, open-label, parallel-group, phase III clinical study. A total of 157 patients were recruited at 14 centers with 147 patients meeting the inclusion/exclusion criteria and randomized to either BP (*n* = 71) or EP (*n* = 76) treatment arms. A non-inferior response rate (RR) in the BP arm, analyzed by intent-to-treat analysis according to Response Evaluation Criteria in Solid Tumors version 1.0 criteria, was used as the primary endpoint. The secondary endpoints were progression-free survival (PFS) and overall survival (OS).

**Results:**

In the BP arm, one patient had a complete response, 41 had a partial response (PR), and 17 had stable disease (SD). In the EP arm, 35 patients had PR and 28 had SD. The RR in the BP arm was non-inferior to the EP regimen in patients with ES-SCLC (BP: 59.2 %, EP: 46.1 %, difference: 13.1 %, 90 % two-sided confidence interval: -0.3–26.5, meeting the predefined non-inferiority criterion of -15.0 %). No significant differences in OS or PFS were observed between the treatment arms. Hematologic toxicities, including grade 3/4 anemia and thrombocytopenia, were significantly more prevalent in the BP arm than the EP arm.

**Conclusions:**

The RR to the BP regimen was non-inferior to the EP regimen in chemotherapy- and radiotherapy-naïve patients with previously untreated ES-SCLC. Hematologic toxicities were significantly more prevalent in the BP group, indicating that BP should be used with care, particularly in patients with a poor performance status. Further studies assessing PFS and OS are required to validate the superiority of the BP regimen.

**Trial registration:**

ClinicalTrials.gov identifier NCT00826644. Date of Registration: January 21, 2009.

## Background

Lung cancer is one of the leading causes of cancer-related death worldwide [1–3]. Small cell lung cancer (SCLC) accounts for up to 20 % of all new cases of lung cancer and deaths [3, 4]. Compared to non-small cell lung cancer (NSCLC), SCLC is generally more aggressive, with decreased doubling times and faster growth rates. Moreover, early widespread metastasis is a recognized feature of SCLC [5]. Extensive-stage SCLC (ES-SCLC) refers to SCLC metastasis to distant body regions. Since the mid-1980s, no significant improvement in the survival of patients with ES-SCLC has been observed; the median overall survival (OS) is estimated at approximately 10 months [6–10]. Currently, a two-drug combination of platinum and etoposide at doses associated with at least moderate toxic effects is most commonly used to treat ES-SCLC [11]. The overall response rates of 50 %–80 % and complete response rates of 0 %–30 % have been reported with this treatment approach [12, 13].

To date, a number of pharmacological agents have been developed for the treatment of NSCLC. However, no novel chemotherapeutic combinations have demonstrated superior efficacy to etoposide/cisplatin (EP), a standard treatment regimen, in the treatment of SCLC over the past decade, although irinotecan/cisplatin (IP) has been reported as an effective combination regimen [10]. Belotecan {7-[2(N-isopropylamino) ethyl]-(20S)-camptothecin} is a newly developed camptothecin analogue. According to two multi-center phase IIa studies, belotecan monotherapy is an effective modality for the treatment of SCLC in chemotherapy-naïve patients [14, 15]. Moreover, multi-center phase II studies have reported response rates (RR) higher than 70 % and OS greater than 10 months in patients with ES-SCLC receiving belotecan/cisplatin (BP) as a first-line treatment regimen [16–18].

On the basis of the above mentioned information, we conducted a multi-center, randomized, open-label, parallel-group, phase III clinical study to compare the efficacy and safety of BP and EP regimens in chemotherapy- and radiotherapy-naïve patients with previously untreated ES-SCLC.

## Methods

### Study patients

Patients who met all of the following inclusion criteria were enrolled in this trial: (1) aged between 19 and 80 years, (2) histologically or cytologically proven ES-SCLC, (3) no past history of chemotherapy or radiotherapy, (4) ≥1 measurable disease according to the Response Evaluation Criteria in Solid Tumors (RECIST) criteria version 1.0, (5) Eastern Cooperative Oncology Group (ECOG) performance status (PS) of ≤2, (6) a life expectancy of ≥12 weeks, (7) adequate organ function [absolute neutrophil count (ANC) ≥1,500/mm^3^, platelet count ≥100,000/mm^3^, hemoglobin ≥9.0 g/dL, total bilirubin level ≤1.5 mg/dL, aminotransferase ≤2-fold upper limit of normal (ULN) or ≤3-fold ULN if demonstrable liver metastases, alkaline phosphatase ≤2-fold ULN, and creatinine ≤1.5 mg/dL or creatinine clearance ≥60 mL/min].

The exclusion criteria were as follows: (1) severe bacterial infection, (2) malignancies other than basal cell skin cancer or cervical carcinoma *in situ*, (3) brain metastases, (4) women with child-bearing potential, and (5) women who are pregnant or breast-feeding.

The study was approved by the Institutional Review Board (IRB) of each medical institution. All patients provided a written informed consent. The current study was registered with ClinicalTrials.gov (Identifier: NCT00826644).

### Dosing rationale and schedule

Patients were randomized to either EP or BP treatment arms and stratified according to ECOG PS (0-1 vs. 2) and age (<65 vs. ≥65 years) at a ratio of 1:1. A phase I study was conducted to determine the maximum tolerated dose (MTD), toxicity, and dose-limiting toxicity of BP; it showed that the MTD and recommended dose for phase II studies was 0.5 mg/m^2^ on days 1–4 in combination with 60 mg/m^2^ cisplatin on day 1 at a 3-week interval [19]. The BP regimen consisted of intravenous belotecan 0.5 mg/m^2^ mixed with 100 mL of 5 % dextrose over 30 min on day 1–4 and intravenous cisplatin 60 mg/m^2^ on day 1 of 3-week cycles. The EP regimen consisted of etoposide 100 mg/m^2^ on days 1, 2 and 3 and cisplatin 60 mg/m^2^ on day 1 of 3-week cycles. Both regimens required hydration and administration of antiemetic drugs.

On day 1, the patients were treated if they showed an ANC ≥1,500/mm^3^, platelet count ≥100,000/mm^3^, and creatinine clearance ≥60 mL/min. In addition, the patients were given recombinant human granulocyte colony-stimulating factor (G-CSF) to improve ANC according to clinical judgment. Subsequent treatments were delayed on a weekly basis until recovery of ANC in cases of G-CSF treatment failure. The patients dropped out of the study if the treatment was delayed by more than 2 weeks. With respect to the dose of the subsequent treatment, the patients were given unadjusted treatment doses upon recovery of ANC to ≥1,500/mm^3^ and a platelet count ≥100,000/mm^3^. In the patients achieving a recovery of ANC to 1,000–1,500/mm^3^ and a platelet count to 75,000–100,000/mm^3^, subsequent treatment doses were reduced by 20 %. In patients with ANC <500/mm^3^, platelet count <25,000/mm^3^, or febrile neutropenia during treatment, subsequent treatment doses were reduced by 20 %. Cisplatin doses were not adjusted in patients with decreases in creatine clearance to ≥60 mL/min from baseline. Cisplatin doses were reduced by 50 % in patients with creatinine clearance of 30–60 mL/min. Cisplatin was discontinued in patients with decreases in creatinine clearance to ≤30 mL/min.

### Patient evaluation

The patients were evaluated at baseline based on their medical history, physical examination, imaging studies, complete blood counts, and serum biochemistry. The response was assessed by computed tomography (CT) every two treatment cycles at follow-up visits. After the completion of chemotherapy, CT scans were performed every three months until evidence or suspicion of disease progression. The treatment response was centrally evaluated independently according to RECIST version 1.0 criteria as follows: (1) complete response (CR), disappearance of all clinical and radiological evidence of the tumor; (2) partial response (PR), decrease of 30 % or more in the sum of longest diameters of all target measurable lesions; (3) progressive disease (PD), increase of more than 20 % of the sum of longest diameters of all target measurable lesions or the appearance of new lesions; and (4) stable disease (SD), all other circumstances. The patients eligible for response evaluation were evaluated at a minimum interval of 4 weeks to confirm CR or PR. Adverse events (AEs) were graded according to the NCI-CTCAE, version 3.0 (http://ctep.cancer.gov/protocolDevelopment/electronic_applications/docs/ctcaev3.pdf).

### Efficacy endpoints

Per-protocol (PP) population was defined patients who completed two cycles of chemotherapy in accordance with the protocol. Modified Intent-to-treat (mITT) population was defined as patients randomized and treated with at least one cycle of chemotherapy. mITT analysis was used to assess non-inferiority of RR in the BP arm as the primary endpoint according to RECIST 1.0 criteria. The secondary endpoints were progression-free survival (PFS) and OS. Subgroup analysis of the mITT population was performed to assess the associations between RR and ECOG PS, age, and body weight.

### Safety endpoints

The safety population comprised all patients eligible for safety analysis. The occurrence of toxicities and adverse effects were evaluated throughout the study period through the measurement of vital signs, physical examination, and clinical laboratory tests. The safety endpoints were adverse drug reactions (ADRs), serious adverse events (SAEs), and treatment-related death (TRD).

### Statistical analyses

To determine the sample size, we estimated an RR of 71 % for the BP arm [14, 15, 19] and 66 % for the EP arm [6, 13, 20], considering a non-inferiority margin of −15 % at a power of 80 % with a one-sided error (α) = 0.05 [21]. In addition, we assumed a dropout rate of 10 % during the follow-up period. Thus, we aimed to enroll a total of 150 patients (*n* = 150) in the present study.

The baseline and clinical characteristics of the patients were expressed as median and range. The Student’s *t*-test and chi-square test were used to compare two treatment arms, as appropriate. Two-sided 90 % confidence intervals (CIs) were calculated for the difference in the RR between the two treatment arms without corrections for continuity, as described by Newcombe [21]. The Cochran–Mantel–Haenszel (CMH) test was performed in patients who met the criteria for non-inferiority with consideration of the stratification factors. Thus, we attempted to analyze the difference in RR between the two treatment arms using Fisher’s exact test. PFS was defined as the time from randomization to clinical or radiological progression or death. OS was defined as the time from randomization to death from any cause. If the patient was lost to follow up or an event (disease progression or death) did not occur until study termination, the patient was censored at the time of last contact. PFS and OS were analyzed using the Kaplan–Meier methods.

## Results

### Patient groups

**Fig. 1:**
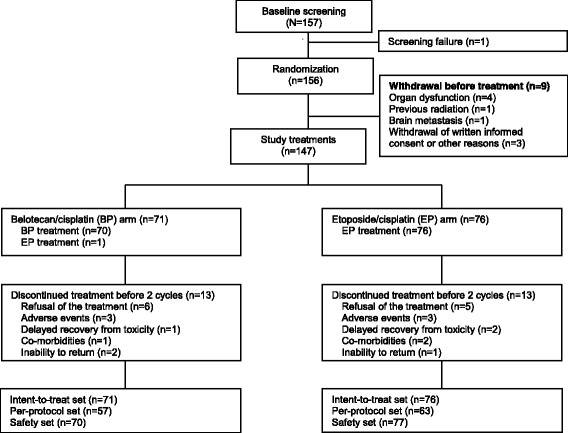
Disposition of the study patients. Of the 157 patients we recruited at a total of 14 centers, 147 met inclusion/exclusion criteria and then randomized to either the BP arm (*n* = 71) or the EP arm (*n* = 76). In the BP arm, the number of the patients of the mITT set, the PP set and the safety set were 71, 57 and 70, respectively. In the EP arm, these values were 76, 63 and 77 in the corresponding order

A total of 157 patients were recruited at 14 centers across Korea from January 2009 to January 2013. Of these, 147 patients met the inclusion/exclusion criteria of this study and were randomized to the BP (*n* = 71) or EP (*n* = 76) arms. The number of the patients in the mITT, PP, and safety populations were 71, 57, and 70 in the BP arm and 76, 63, and 77 in the EP arm, respectively (Fig. 1).

### Patient baseline and clinical characteristics

No significant differences in the median age, median male-to-female ratio, median body surface area, or median ECOG PS were observed between the treatment arms (Table 1). A significant difference in the median body mass index was observed between the treatment arms (*p* < 0.05).

**Table 1:** Patient demographics of modified intent-to-treat population

Characteristic	Belotecan/Cisplatin (*n* = 71)		Etoposide/Cisplatin (*n* = 76)		*p* value
	No.	%	No.	%	
Ages, years					.565
Median	67.0		66.5		
Range	29–91		46–80		
Sex					.751
Male	62	87.3	65	85.5	
Female	9	12.7	11	14.5	
Body mass index, kg/m^2^					.004
Median	23.5		22.0		
Range	15.1–30.4		15.0–30.8		
Body surface area, m^2^					.055
Median	1.71		1.65		
Range	1.30–1.99		1.26–2.06		
ECOG performance status					.200
0	23	32.4	20	26.3	
1	37	52.1	35	46.1	
2	11	15.5	21	27.6	

Abbreviation: *ECOG* Eastern cooperative oncology group

The proportion of the patients receiving more than four cycles of chemotherapy was 67.1 % in the BP arm and 63.7 % in the EP arm. The mean number of chemotherapy cycles was 3.9 in the BP arm and 4.1 in the EP arm. In the BP arm, the mean delivered dose of belotecan and cisplatin was 0.45 mg/m^2^ (90.0 % of planned dose) and 56.8 mg/m^2^ (94.6 % of planned dose), respectively. In the EP arm, the mean delivered dose of etoposide and cisplatin was 93.8 mg/m^2^ (93.8 % of planned dose) and 58.6 mg/m^2^ (97.7 % of planned dose), respectively. The relative dose intensity (RDI) was calculated by dividing the intensity of the delivered dose by that of the standard dose. A significantly lower RDI was observed in the BP arm than that in the EP arm (0.79 ± 0.14 vs. 0.86 ± 0.13, *p* = 0.001).

### Efficacy endpoints

#### Response rates

**Fig. 2:**
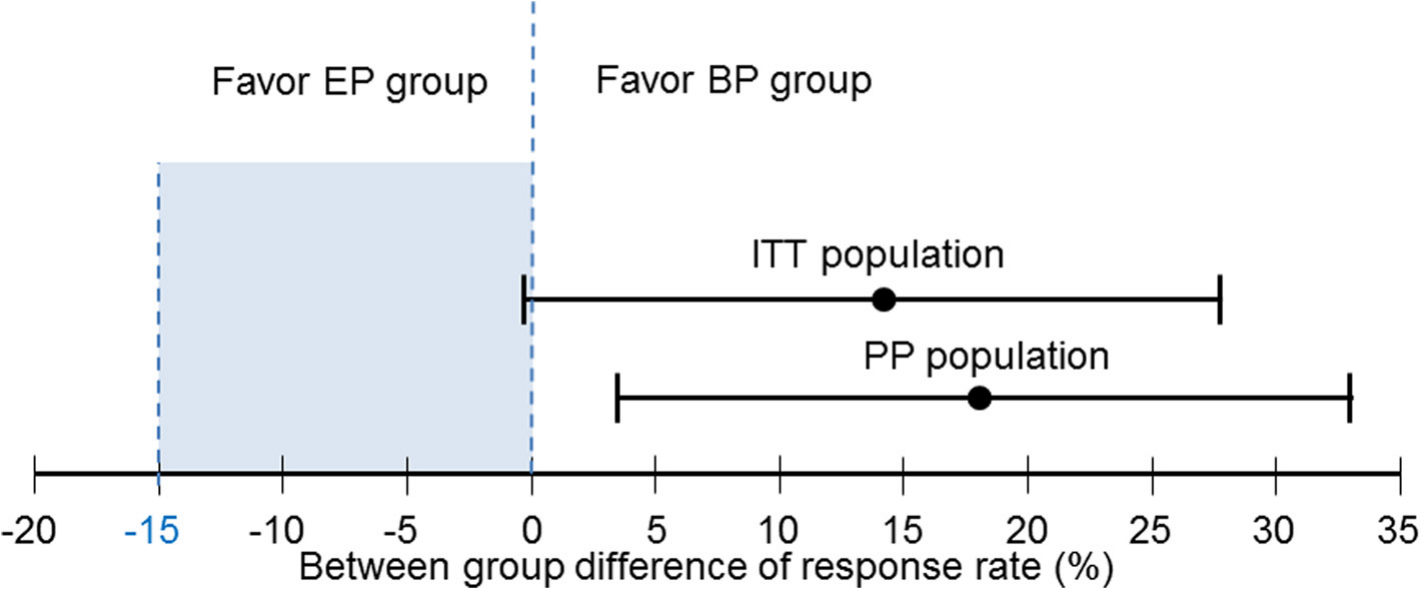
Efficacy endpoints of modified intent-to-treat (mITT) and per protocol (PP) population. The BP group is not inferior to the EP group with regards to the response rate (between group difference 13.1 %, 90 % two-sided confidence interval -0.3 to 26.5, meeting the predefined non-inferiority criterion of -15.0 %) in mITT population. The response of the BP group was superior in PP population

According to the mITT analysis, the RR was 59.2 % in the BP arm and 46.1 % in the EP arm (Table 2 & Fig. 2). The lower limit of the two-sided 90 % CI was greater than the non-inferiority margin (−0.3 vs. −15.0). This indicates that the BP regimen was non-inferior to the EP regimen with respect to the RR in patients with ES-SCLC.

**Table 2:** Best overall response of modified intent-to-treat and per protocol population

*Modified intent-to-treat population*							
Response	BP (*n* = 71)		EP (*n* = 76)		Difference	90 % CI	*P* value by CMH test
	No.	%	No.	%			
CR	1	1.4	0	0			
PR	41	57.7	35	46.1			
SD	17	23.9	28	36.8			
PD	4	5.6	5	6.6			
NE	8	11.3	8	10.5			
CR + PR	42	59.2	35	46.1	13.1	−0.3 to 26.5	.214
CR + PR + SD	59	83.1	63	82.9	0.2	−10.0 to 10.4	.826
*Per protocol population*							
Response	BP (*n* = 57)		EP (*n* = 63)		Difference	90 % CI	*P* value by CMH test
	No.	%	No.	%			
CR	1	1.8	0	0			
PR	40	70.2	34	54.0			
SD	9	15.8	21	33.3			
PD	3	5.3	3	4.8			
NE	4	7.0	5	7.9			
CR + PR	41	71.9	34	54.0	18.0	3.7 to 32.2	.061
CR + PR + SD	50	87.7	55	87.3	0.4	−0.95 to 10.4	.978

Abbreviation: *BP* belotecan/cisplatin, *EP* etoposide/cisplatin, *CI* Confidence interval, *CMH* Cochran-Mantel-Haenzel, *CR* complete response, *PR* partial response, *SD* stable disease, *PD* progressive disease, *NE* non-evaluable cases

According to the PP analysis, an 18.0 % difference in the RR was demonstrated between the two treatment arms (90 % CI: 3.7–32.3). In addition, no significant differences in the RRs between the two treatment arms in either the mITT or PP groups were observed according to the CMH test.

#### Overall survival and progression-free survival

**Fig. 3:**
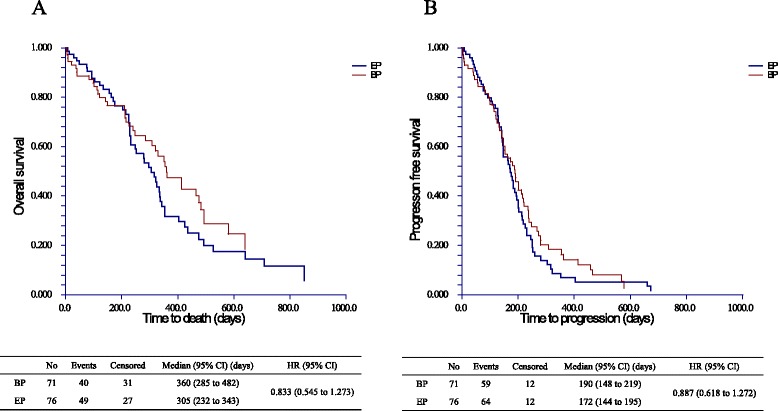
Overall survival and progression-free survival on Kaplan–Meier analysis. (**a**) There were no significant differences in the OS and PFS between the two treatment arms. That is, the median OS was 360 days (95 % CI: 285–482) in the BP arm and 305 days (95 % CI: 232–343) in the EP arm (Log-Rank *p* = 0.210). (**b**) The median PFS was 190 days (95 % CI: 148-219) in the BP arm and 172 days (95 % CI: 144–195) in the EP arm (Log-Rank *p* = 0.369)

No significant differences in OS or PFS were observed between the treatment arms. The median OS was 360 days (95 % CI: 285–482) in the BP arm and 305 days (95 % CI: 232–343) in the EP arm (Log-Rank *p* = 0.210, Fig. 3). In addition, the median PFS was 190 days (95 % CI: 148–219) in the BP arm and 172 days (95 % CI: 144–195) in the EP arm (Log-Rank *p =* 0.369, Fig. 3).

### Subgroup analysis

In the BP arm, the RR was 63.3 % in patients with ECOG PS 0-1 (*n* = 60) and 36.4 % in those with ECOG PS 2 (*n* = 11). In the EP arm, the RR was 52.7 % in patients with ECOG PS 0–1 (*n* = 55) and 28.6 % in those with ECOG PS 2 (*n* = 21). In addition, the RR was 66.7 % in patients aged ≤65 years (*n* = 30) and 53.7 % in those aged ≥65 years (*n* = 41) in the BP arm. In the EP arm, the RR was 57.8 % in patients aged ≤65 years (*n* = 33) and 37.2 % in those aged ≥65 years (*n* = 43). Furthermore, the RR was 60.0 % in patients weighing less than 62.5 kg (*n* = 30) and 58.5 % in those weighing more than 62.5 kg (*n* = 41) in the BP arm. In the EP arm, the RR was 45.2 % in patients weighing less than 62.5 kg (*n* = 42) and 47.1 % in those weighing more than 62.5 kg (*n* = 34).

### Safety endpoints

In the present study, all 147 included patients were eligible for safety analysis. No significant differences in ADRs or TRD were observed between the treatment arms (98.6 % vs. 92.2 %, *p* = 0.119 and 12.9 % vs. 10.4 %, *p* = 0.797, respectively). However, the incidence of SAEs was significantly higher in the BP arm (42/70 cases, 60.0 %) than those in the EP arm (31/77 cases, 40.3 %, *p* = 0.021). Hematologic toxicities were more prevalent in this study than non-hematologic toxicities. Grade 3/4 anemia (34.3 %) and thrombocytopenia (54.3 %) were significantly more prevalent in the BP arm than those in the EP arm. No significant difference in the incidence of non-hematologic toxicities was observed between the treatment arms. Grade 3/4 infection and hyponatremia occurred in approximately 10 % of the patients in both treatment arms. Nearly all the non-hematologic toxicities were of grade 1/2 severity and could be treated successfully (Table 3).

**Table 3:** Hematologic and non-hematologic toxicities of safety population

	BP Group (*n* = 70)					EP Group (*n* = 77)					*P* ^†^
	Grade^a^, *n* (%)					Grade^a^, *n* (%)					
	1	2	3	4	≥3	1	2	3	4	≥ 3	
Febrile neutropenia	0(0.0)	0(0.0)	7(10.0)	4(5.7)	11(15.7)	0(0.0)	0(0.0)	6(7.8)	0(0.0)	6(7.8)	0.196
Anemia	3(4.3)	30(42.9)	21(30.0)	3(4.3)	24(34.3)	13(16.9)	33(42.9)	10(13.0)	0(0.0)	10(13.0)	0.003
Leukopenia	3(4.3)	13(18.6)	23(32.9)	19(27.1)	42(60.0)	5(6.5)	17(22.1)	25(32.5)	10(13.0)	35(45.5)	0.098
Neutropenia	1(1.4)	3(4.3)	11(15.7)	43(61.4)	54(77.1)	2(2.6)	9(11.7)	14(18.2)	38(49.4)	52(67.5)	0.204
Thrombocytopenia	2(2.9)	14(20.0)	21(30.0)	17(24.3)	38(54.3)	13(16.9)	4(5.2)	7(9.1)	6(7.8)	13(16.9)	< 0.001
Anorexia	7(10.0)	13(18.6)	1(1.4)	0(0.0)	1(1.4)	6(7.8)	9(11.7)	1(1.3)	0(0.0)	1(1.3)	1.000
Nausea	12(17.1)	12(17.1)	2(2.9)	0(0.0)	2(2.9)	10(13.0)	7(9.1)	2(2.6)	0(0.0)	2(2.6)	1.000
Vomiting	8(11.4)	5(7.1)	1(1.4)	0(0.0)	1(1.4)	4(5.2)	3(3.9)	0(0.0)	0(0.0)	0(0.0)	0.473
Weight loss	0(0.0)	0(0.0)	1(1.4)	0(0.0)	1(1.4)	0(0.0)	3(3.9)	1(1.3)	0(0.0)	1(1.3)	1.000
Anxiety	1(1.4)	2(2.9)	2(2.9)	0(0.0)	2(2.9)	3(3.9)	3(3.9)	0(0.0)	0(0.0)	0(0.0)	0.473
Diarrhea	14(20.0)	7(10.0)	2(2.9)	0(0.0)	2(2.9)	8(10.4)	2(2.6)	1(1.3)	0(0.0)	1(1.3)	0.605
Fatigue	8(11.4)	11(15.7)	4(5.2)	0(0.0)	4(5.2)	18(23.4)	12(15.6)	3(3.9)	0(0.0)	3(3.9)	0.498
Infection	0(0.0)	0(0.0)	9(12.9)	3(4.3)	13(18.6)	0(0.0)	4(5.2)	5(6.5)	0(0.0)	10(13.0)	0.577
Hepatic dysfunction	1(1.4)	5(7.1)	2(2.9)	0(0.0)	2(2.9)	4(5.2)	3(3.9)	4(5.2)	2(2.6)	6(7.8)	0.621
Hyperglycemia	0(0.0)	4(5.7)	2(2.9)	0(0.0)	2(2.9)	0(0.0)	3(3.9)	4(5.2)	1(1.3)	5(6.5)	0.446
Hyponatremia	1(1.4)	0(0.0)	6(8.6)	6(8.6)	12(17.1)	0(0.0)	1(1.3)	6(7.8)	3(3.9)	9(11.7)	0.631
Hyperkalemia	0(0.0)	0(0.0)	2(2.9)	0(0.0)	2(2.9)	0(0.0)	1(1.3)	3(3.9)	0(0.0)	3(3.9)	1.000
Hypokalemia	0(0.0)	0(0.0)	3(4.3)	0(0.0)	3(4.3)	1(1.3)	0(0.0)	1(1.3)	0(0.0)	1(1.3)	0.347

^a^Grade means the maximum grade of toxicity. Grade 5 toxicities were developed by infection (1 patient in BP arm and 5 patients in EP arm) and disease progression (2 patients in EP arm)

†*P* value was calculated by Fisher’s exact test for grade ≥ 3 toxicity

## Discussion

This is the first study to evaluate the non-inferiority of the BP regimen compared to the EP regimen as a first-line treatment in patients with previously untreated ES-SCLC. Compared to the EP regimen, the RR of the BP regimen was non-inferior in the mITT population and better in the PP population. Grade 3/4 anemia and thrombocytopenia were more prevalent in the BP arm than those in the EP arm. In addition, the RDI was significantly lower in the BP arm than that in the EP arm. However, no significant differences in OS or PFS were observed between the treatment arms.

Over recent decades, EP has been considered the gold-standard treatment for ES-SCLC. In 2002, a phase III trial was conducted by the Japan Clinical Oncology Group; it demonstrated the superiority of IP over EP in patients with ES-SCLC [10]. However, two subsequent randomized phase III trials failed to confirm the superiority of IP over EP in North American and Australian populations [7, 8]. Noda et al. reported that the RR and median OS were 65 % and 12.8 months in the IP arm, respectively [10]. Our results were consistent with previous studies, demonstrating a similar efficacy based on an RR of 60 % and a median OS of 12.9 months. However, we found a higher proportion of patients with ECOG PS 2 than the study by Noda et al. (16 % vs. 8 %). Moreover, the RR in the present study was approximately 10 % lower than previous phase II trials enrolling a smaller number of patients [16–18]. To date, Lim et al. have reported the highest RR (73.8 %) with the use of the BP regimen [18]. Lee et al. reported a median PFS of 6.9 months and a median OS of 19.2 months in patients receiving the BP regimen [16]. In the current study, the degree of the difference in the RR between the two treatment arms was higher in the PP population than the mITT population (18.0 % vs. 13.1 %). Moreover, our results also showed a slightly higher RR in patients with ECOG PS 0–1 (63.3 %) and those aged ≤65 years (66.7 %). These results indicate that the BP regimen may have a greater utility in younger patients with a good performance status.

In the present study, favorable rates of non-hematologic toxicities were observed in the BP arm according to the safety analysis. We found that 2.9 % of the patients in the BP arm developed grade 3/4 diarrhea; this may be considerably lower than the incidence of non-hematologic toxicities reported in the IP arm of previous trials (16 %–19 %) [8, 10]. In the BP arm, neutropenia was one of the most frequent hematologic toxicities, 77 % of which were of grade 3 or 4 severity. Grade 3/4 febrile neutropenia occurred in 15.7 % of patients in the BP arm. Of these, one patient died of pneumonia. No significant differences in the incidences of neutropenia or febrile neutropenia were observed between the treatment arms. In the EP arm, there were five cases of treatment-related deaths due to pneumonia or sepsis. However, grade 3/4 anemia (34.3 % vs. 13.0 %, *p* = 0.003) and thrombocytopenia (54.3 % vs. 16.9 %, *p* < 0.001) were significantly more prevalent in the BP arm than the EP arm. The prophylactic use of G-CSF or 5 %–10 % reductions in chemotherapeutic doses may be considered to prevent hematologic toxicities. In the present study, the mean RDI was 7 % lower in the BP arm than that in the EP arm (0.79 ± 0.14 vs. 0.86 ± 0.13). A previous phase II study found that more than half of the patients presented with grade 4 neutropenia and reported an RDI of 70.1 % in the belotecan regimen and 83.0 % in the cisplatin regimen. As a result of the findings of this trial, the recommended BP regimen dose of BP was reduced by 25 % (0.5 mg/m^2^ for 3 days) [17].

A first limitation of this study is the non-inferiority design of this trial. Because the experimental arm demonstrated a higher hematological toxicity, superior results are required to justify the use of the experimental treatment. Because the analysis of the PP group demonstrated a better response rate with the BP regimen than the EP regimen, further studies are required to validate the superior efficacy of the BP regimen. Second limitation is the significance level in the statistical design. Although setting the significance level for one-sided, non-inferiority trials is recommended at 0.025, we chose the alpha level at 0.05 to accomplish the trial within reasonable time span. Third, the ORR which was primary endpoint of this study may not be sufficiently correlated to patient’s outcome. We selected ORR instead of OS because this study was conducted with small sample size. Fourth, the ITT set of this study was mITT population as 9 randomized patients are excluded because they did not receive the randomly assigned regimen. They were three subjects who withdrew informed consents and six who did not meet inclusion criteria. If a subject who actually did not receive any treatment is included as a subject who received treatment, then it indicates very little about the efficacy of the treatment [22]. So we defined mITT population as patients randomized and treated with at least one cycle of chemotherapy. Fifth, the interpretation of subgroup analyses for ECOG PS score, age and body weight was limited because of the issue of multiple comparisons. Finally, as the open-label nature of this study could be regarded as a flaw in a non-inferiority study. However, because these two regimens have different infusion protocols, we were unable to design this study as a blinded trial.

## Conclusions

The results of this trial indicate that the RR with the BP regimen is non-inferior to the EP regimen in chemotherapy- and radiotherapy-naïve patients with previously untreated ES-SCLC. However, hematologic toxicities, including anemia and thrombocytopenia, were more prevalent in the BP arm. These findings strongly suggest that clinicians should be careful in prescribing the BP regimen to elderly patients or those with a poor performance status for the purpose of preventing hematologic toxicities. Further studies evaluating PFS and OS are required to validate the superiority of the BP regimen.

## Abbreviations

ADR: Adverse drug reactions
AEs: Adverse events
ANC: Absolute neutrophil counts
BP: Belotecan and cisplatin
CI: Confidence interval
CMH: Cochran-mantel-haenzel
CR: Complete response
ECOG: Eastern cooperative oncology group
EP: Etoposide and cisplatin
ES-SCLC: Extensive-stage small cell lung cancer
G-CSF: Granulocyte colony-stimulating factor
HR: Hazards ratio
IP: Irinotecan and cisplatin
IRB: Institutional review board
mITT: Modified intent-to-treat
MTD: Maximum tolerated dose
NSCLC: Non-small cell lung cancer
OS: Overall survival
PD: Progressive disease
PFS: Progression-free survival
PP: Per-protocol
PR: Partial response
PS: Performance status
RDI: Relative dose-intensity
RECIST: Response evaluation criteria in solid tumors
RR: Response rate
SAE: Serious adverse events
SCLC: Small cell lung cancer
SD: Stable disease
TRD: Treatment-related death
ULN: Upper limit of normal
